# A comparison between chemical and gas hypoxia as models of global ischemia in zebrafish (*Danio rerio*)

**DOI:** 10.1002/ame2.12132

**Published:** 2020-08-10

**Authors:** Kaitlyn M. Marino, Emani R. Silva, James A. Windelborn

**Affiliations:** ^1^ Department of Biology Washington College Chestertown MD USA; ^2^ Department of Psychology Washington College Chestertown MD USA

**Keywords:** hypoxia, hypoxic‐ischemia, ischemia

## Abstract

**Background:**

Zebrafish models for neurovascular diseases offer new methods for elucidation of molecular pathways to tissue damage. External fertilization and high fecundity provide opportunities for transgenics and other forms of genetic manipulation that are more accessible than offered by mammalian models of disease. Furthermore, behavioral analyses of zebrafish allow for connection of molecular pathways to organismal outputs such as locomotion, learning, and memory. Unfortunately, a zebrafish model of hypoxia‐ischemia has been slow to catch on, possibly due to hypoxia exposure protocols that are challenging to reproduce and result in high mortality.

**Methods:**

In this study, we have introduced a predictable and simple method of hypoxia induction, the addition of sodium sulfite to aquarium water. The effects of this treatment on zebrafish locomotion were compared to those of zebrafish exposed to hypoxia induced by nitrogen gas bubbling, a method used in previous reports.

**Results:**

We found that hypoxia induced by sodium sulfite significantly impaired locomotion in the hours following treatment, and its effects did not differ from those caused by nitrogen gas hypoxia.

**Conclusion:**

These results indicate that hypoxia by sodium sulfite represents an effective and easily reproducible method for the study of hypoxia‐ischemia in zebrafish.

## 1 INTRODUCTION

Zebrafish models for neurovascular diseases have been developed over the past decade,^1^ and several advantages of zebrafish as a laboratory model have been reported. The foremost one is external fertilization that provides up to 200 embryos per instance that are susceptible to genetic manipulation through techniques including Tet‐On control systems, morpholino antisense targeting, and CRISPR‐Cas9 vector systems.^2^, ^3^, ^4^ Researchers can thus perform high‐throughput genetic screens or establish transgenic lines in relatively few generations compared to rodent models.

Zebrafish also offer the ability to apply many of the same neurobehavioral assays used in rodents.^5^ Assays include measurement of visual discrimination, social behavior, novel object recognition, anxiety, and conditioned place preference.^6^, ^7^, ^8^, ^9^, ^10^, ^11^ Furthermore, the effects of pharmacologic treatments on these behaviors can be simply discerned. For instance, adult fish exposed to an NMDA receptor antagonist, MK‐801, dissolved in tank water either before or after training were found to spend significantly less time in the novel arm of a Y‐maze,^12^ suggesting that both memory acquisition and consolidation are under glutamatergic control. For many decades, manipulation of glutamatergic systems has been of interest for the development of treatments for cerebral ischemia, and studies like this show that zebrafish provide a model system capable of examining responses spanning molecular and behavioral approaches.^12^

Two general methods for the formation of cerebral ischemia in zebrafish have been developed.^13^, ^14^ The first, and more broadly used, method is the hypoxia‐ischemia model in which fish are exposed to hypoxic conditions (dissolved oxygen <1mg/L) by bubbling tank water with nitrogen gas. The injury endpoint is determined by cessation of locomotion and slowing of opercular movements. Using these criteria, mortality was 61% and extensive brain damage was detected in subjects up to 24, but not 48, hours after hypoxia.^13^, ^15^ Interestingly, measurements of locomotion such as distance travelled, turn angle, and meandering time revealed a difference between hypoxic and control fish at 1 hour, but not 3‐48 hours, after injury.^15^

Our goals for this study included reducing mortality measured in previous literature while maintaining the behavioral effects of hypoxia demonstrated in those reports. We predicted that modifying our hypoxic chamber to eliminate aquatic surface respiration would reduce the duration of hypoxia necessary for measurable effects, and thereby reduce the risk of mortality. We also chose to explore a novel method for the induction of hypoxia that may reduce inter‐researcher variability in the application of the treatment. Sodium sulfite is an antioxidant commonly used to scavenge oxygen in manufacturing and industrial processes. For instance, it is applied to dried fruits and shellfish to prevent oxidation and extend shelf life.^16^ It has also been used for decades to prevent corrosion in boiler systems.^17^ Beyond these commercial and industrial applications, sodium sulfite has been used in laboratory settings to create hypoxic conditions in aqueous solutions. For instance, sodium sulfite‐induced hypoxia in *Caenorhabditis elegans* was developed to avoid the potential toxicity of other hypoxia‐inducing agents that contain heavy metals.^18^ In that study, subjects exposed to sodium sulfite showed the same severity of injury as those exposed to physical hypoxia maintained by deoxygenated media in a sealed hypoxia chamber. They also indicated that treatment with 0.5 g/L sodium sulfite resulted in reduction of dissolved oxygen in aqueous buffer to below 0.3 mg/L for at least 48 hours.

In zebrafish, sodium sulfite has been utilized as an antioxidant to protect against toxins causing skin epithelium damage. In one study, 1 mM (0.126 mg/L) sodium sulfite reduced skin cell death caused by exposure of 4 dpf larvae to manufactured nanoparticles over a 24‐hour period.^19^ The authors reported no adverse effects of sodium sulfite exposure.

We have applied sodium sulfite as a method of chemical hypoxia‐ischemia in zebrafish. Because locomotor deficits are the most established parameters of hypoxia‐ischemia in laboratory zebrafish, we chose to compare the effects of chemical hypoxia‐ischemia by sodium sulfite to the effects of the more established protocol of hypoxia‐ischemia by nitrogen gas bubbling. Our results indicate that in addition to matching the locomotor effects of nitrogen gas hypoxia, hypoxia by sodium sulfite offers several benefits that make it a simple and reproducible method.

## 2 METHODS

### 2.1 Animal care and use

All procedures were approved by the Washington College Institutional Animal Care and Use Committee (protocol SU17‐002). Zebrafish were purchased from Aquatic Research Organisms and were housed in Washington College's aquaculture center where they were fed twice daily and maintained at 28°C on a 14‐hour light/10‐hour dark cycle. Health checks were performed by staff under the guidance of a consulting laboratory veterinarian at the times of feedings. As described in Table 1, 62 adult fish (>3 months) were exposed to hypoxia in the course of this study. Nine subjects did not recover from exposure to hypoxia (Table 1). An additional 10 subjects were used as normoxic controls without incident. Anesthetics were not administered during treatments to prevent additional hypoxia‐independent depression of respiratory rate.^20^ All subjects were euthanized by rapid chilling, as described by the American Veterinary Medical Association's Panel on Euthanasia.^21^

**TABLE 1:** Survival with chemical vs gas hypoxia

	Surviving/total subjects	% Survival
5‐min chemical hypoxia	30/34	88.2
7‐min chemical hypoxia	1/4	25
7‐min gas hypoxia	4/4	100
10‐min gas hypoxia	26/28	92.9

### 2.2 Behavioral observations

Fish were removed from their home system and placed into an acrylic observation tank. Each tank was 3 inches deep, 6 inches tall, and 10 inches wide with a clear front, opaque white wall and dividers between each tank. Observation tanks were back‐lit, and overhead lighting was turned off, to increase contrast during recording. A 6‐minute observation video was recorded prior to hypoxic treatment (Canon SX530HS). The same procedure was followed at both 2 hours and 24 hours post‐treatment.

### 2.3 Nitrogen gas hypoxia

Filtered, dechlorinated water was added to a 350‐mL polystyrene jar with lid and placed in a water bath set to 28°C. An air stone was added to the nitrogen input. Nitrogen flow was initiated and allowed to bubble for at least 30 minutes, or until the dissolved oxygen level was below 0.6 mg/L. All dissolved oxygen measurements were made using a factory calibrated ProSolo handheld sampling meter with ODO/CT probe (YSI, Yellow Springs, Ohio). The nitrogen input was removed, and a single fish was placed in the chamber with a lid that eliminated all air gaps for 10 minutes. Fish were then returned to their home system water and allowed to recover under observation until balanced swimming was achieved.

### 2.4 Chemical hypoxia

Filtered, dechlorinated water was added to a 350‐mL polystyrene jar. A quantity of 0.5 g/L sodium sulfite (sodium sulfite, anhydrate, Bio Basic, Amherst, New York) was added and allowed to sit until the dissolved oxygen level reached less than 0.6 mg/L. Individual fish were removed from the observation tank and placed into the hypoxia chamber, with a lid that eliminated all space for air, for 5 minutes. Fish were then returned to their home system water and allowed to recover under observation until balanced swimming was achieved. Subjects would have been euthanized by rapid chilling if balanced swimming had not been achieved within 30 minutes following hypoxia.

### 2.5 Behavioral analysis

Video observations were imported into Ethovision XT 14 (Noldus) and analyzed for total distance, velocity, and total time in motion.

### 2.6 Statistical analysis

All statistical analyses were completed in SPSS (IBM) at the significance level of *P* < .05. Error bars in charts represent one standard deviation. Details of individual tests can be found in figure legends and text of Results.

## 3 RESULTS

### 3.1 Sodium sulfite‐induced hypoxia

**FIGURE 1:**
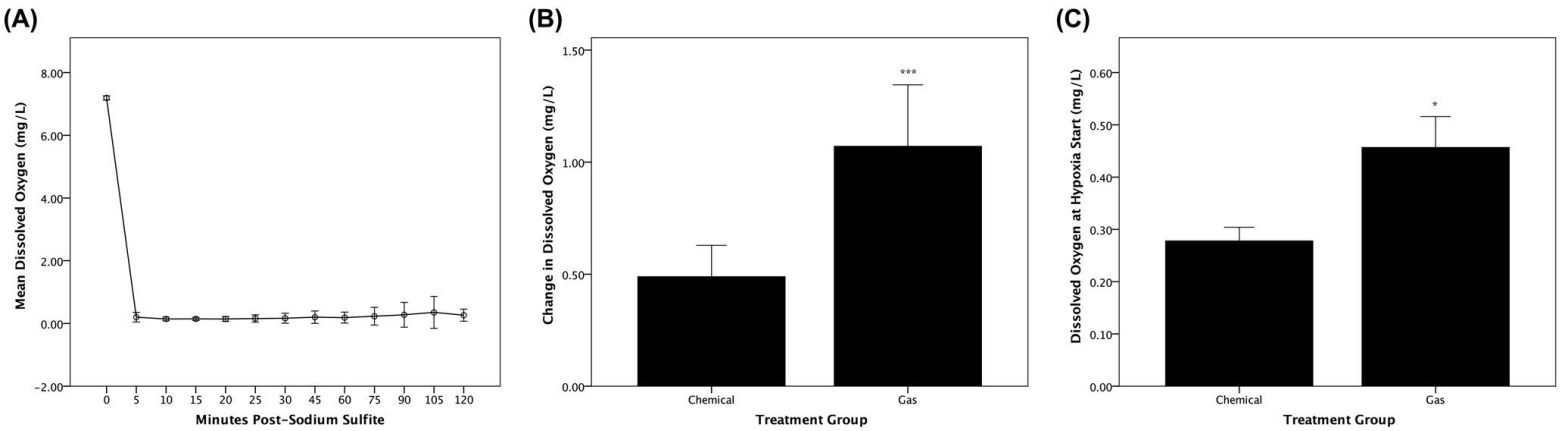
Dissolved oxygen profiles with chemical vs nitrogen gas hypoxia. A, The longevity of hypoxia caused by 0.5 g/L sodium sulfite added to aquarium water was tested by measuring (in the absence of fish) dissolved oxygen levels prior to addition and at 5‐minute intervals following addition of the chemical (n = 3). B, Dissolved oxygen concentration in the hypoxic chamber immediately prior to fish exposure was subtracted from the concentration immediately following exposure to calculate a change in dissolved oxygen during chemical (n = 9) and nitrogen gas (n = 9) hypoxic treatments. ***t = −4.365(16); *P* = .0005. C, Dissolved oxygen concentration in the hypoxic chamber immediately prior to fish exposure is also reported.*t = −2.760(16); *P* = .014

The effectiveness of a single addition of sodium sulfite to a concentration of 0.5 g/L was tested in a hypoxia chamber (Figure 1A). Following addition of sodium sulfite at 0 minutes, the level of dissolved oxygen (DO) in the chamber fell from levels of 7‐8 mg/L to <0.5 mg/L. When left undisturbed, DO concentration remained below 0.5 mg/L for at least 120 minutes. This compares favorably with the effects of continuous bubbling of water in a hypoxia chamber with 100% nitrogen.^22^

The DO concentrations were measured before and after exposure of zebrafish to either chemical (0.5 g/L sodium sulfite for 5 minutes) or gas (100% nitrogen for 10 minutes) hypoxia (Figure 1B). Nitrogen gas bubbling was ceased immediately prior to addition of fish to the hypoxia chamber to avoid development of gas bubble disease in the subjects exposed to nitrogen‐saturated water.^23^ As determined by an independent‐samples *t* test, DO concentrations in chamber water rose significantly more during fish exposure to 10‐minute nitrogen gas hypoxia (1.0711 ± 0.35635) mg/L than to 5‐minute chemical hypoxia (0.4889 ± 0.18196) mg/L; *t* = −4.365(16), *P* = .0005. DO concentrations through the 5‐minute exposure to chemical hypoxia were thus more stable than the 10‐minute exposure to gas hypoxia.

The mean DO concentrations immediately prior to fish exposure to hypoxia are also reported (Figure 1C). The mean DO concentration obtained by bubbling with nitrogen gas (0.46 ± 0.06) mg/L was significantly greater than the mean obtained by addition of 0.5 g/L sodium sulfite (0.28 ± 0.03) mg/L; *t* = −2.760(16), *P* = .014. Along with results reported in Figure 1B, this indicates that fish will be exposed to more severe hypoxia if induced by sodium sulfite rather than nitrogen gas bubbling.

### 3.2 Survival with chemical vs gas hypoxia

As one of our major goals was a reduction in mortality, we calculated survival rates across hypoxic treatments in adult (>3 months) zebrafish. Five‐minute chemical hypoxia and 10‐minute gas hypoxia yielded similar survival (88.2% and 92.9%, respectively). Of 34 zebrafish exposed to 5‐minute chemical hypoxia, 30 survived the trauma, while 26 of 28 fish exposed to 10‐minute gas hypoxia survived (Table 1). Each subject was placed into the hypoxic chamber alone. Durations of hypoxia were shifted to determine whether a dose effect on survival existed. When the duration of chemical hypoxia was raised from 5 minutes to 7 minutes, survival fell to 25% (1/4 fish). In contrast, when the duration of gas hypoxia was lowered from 10 minutes to 7 minutes, survival rose to 100% (4/4 fish).

### 3.3 Locomotor behaviors with 2‐hour recovery

Reduced locomotion was expected within 1‐6 hours following hypoxia,^15^, ^24^ and our chosen post‐hypoxia interval of 2 hours was safely within those limits. Fish were assigned to one of the three possible treatment groups. *Normoxia* fish were kept in normoxic recovery tanks containing 7‐8 mg/L DO throughout the experiment, except when recorded in observation tanks before treatment and 2 hours after treatment. *Chemical* fish were recorded for 6 minutes in observation tanks before hypoxia, exposed to 5‐minute chemical hypoxia, recovered in normoxic recovery tanks for 2 hours, and then were recorded again in observation tanks. *Gas* fish followed the same procedures as *Chemical* fish but were exposed to 10‐minute gas hypoxia rather than to chemical hypoxia.

**FIGURE 2:**
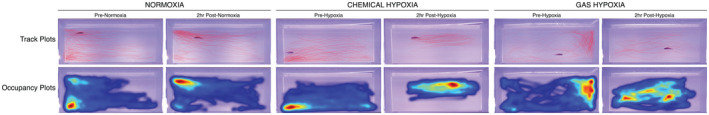
Locomotion plots of adult zebrafish both immediately before and 2 hours after treatment with normoxia or hypoxia. Track plots (top row) and occupancy plots (heat maps, bottom row) were generated by Ethovision XT and overlayed on a reference image for each representative fish. The plots for 3 individual fish are shown (1 fish for each treatment group, with one track calculated pre‐treatment and the other track at 2 hours post‐treatment). These fish were chosen for this figure because their distances travelled and velocities before and after treatment most closely matched the averages displayed in Figure 3

Example tracks for fish both immediately before and 2 hours after treatment with normoxia, chemical hypoxia, or nitrogen gas hypoxia are provided to demonstrate typical changes in locomotion following each treatment (Figure 2). The track plots and occupancy plots from three individual fish are shown in Figure 2. Each fish was recorded immediately before and 2 hours after treatment, and Ethovision XT plotted its location 30 times per second for the 6‐minute observation period.

**FIGURE 3:**
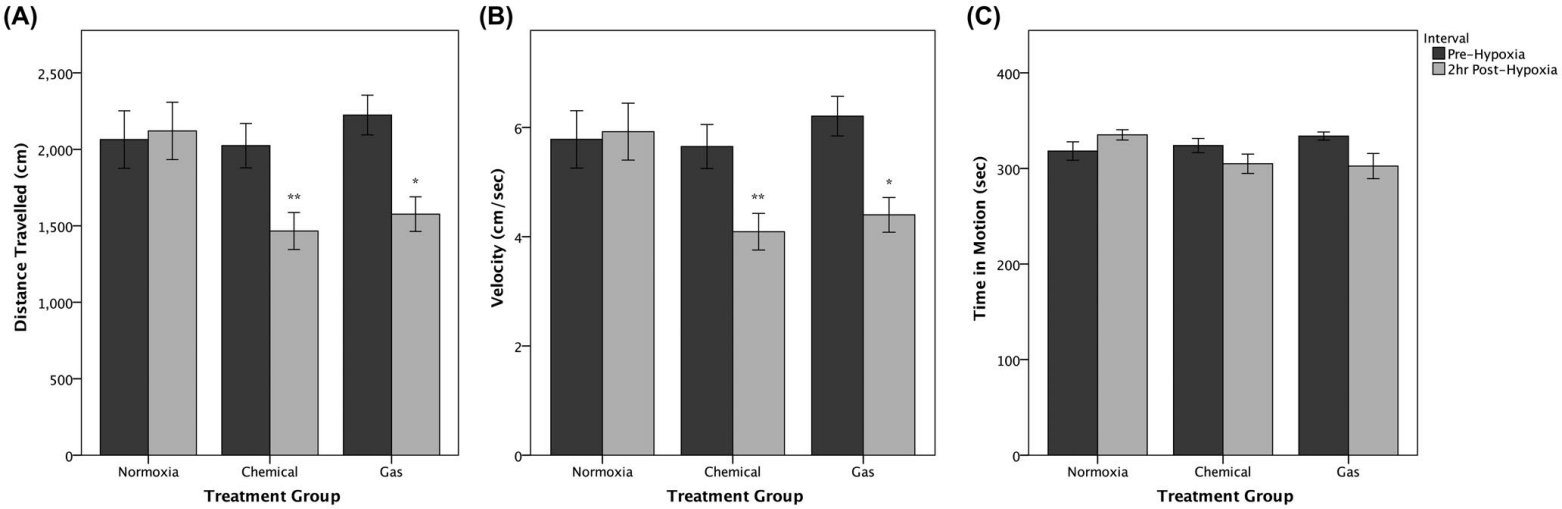
Locomotor behaviors immediately before and 2 hours after chemical vs nitrogen gas hypoxia. Zebrafish were recorded for 360 seconds prior to treatment exposure and recorded again at a post‐treatment interval of 2 hours. Normoxia subjects were not exposed to hypoxia (n = 10), chemical subjects were exposed to 5‐minute hypoxia caused by dissolved sodium sulfite (0.5 g/L; n = 12), and gas subjects were exposed to 10‐minute hypoxia caused by bubbling with nitrogen gas (n = 11). Data are mean ± standard error. (A) Distance travelled and (B) velocity during the 2‐hr post‐hypoxia interval were significantly decreased in both the chemical (*P* = .007) and gas (*P* = .03) groups compared to the normoxia group. C, No main effect for time in motion was detected in either the chemical (*P* = .115) or gas (*P* = .09) hypoxia groups compared to the normoxia group post‐hypoxia

The effects of treatment group and interactions between treatment and recording interval (pre‐hypoxia or 2 hr post‐hypoxia) were examined by two‐way mixed ANOVA.^25^ Statistically significant interactions between the treatment group and observation interval on distance (*F*(2, 30) = 6.618, *P* = .004, partial *η* ^2^ = 0.306), velocity (*F*(2, 30) = 6.506, *P* = .004, partial *η* ^2^ = 0.303), and time in motion (*F*(2, 30) = 4.333, *P* = .022, partial *η* ^2^ = 0.224) were detected (Figure 3). Two hours post‐hypoxia, distance travelled was significantly lower in chemical (*P* = .007) and gas (*P* = .031) treatment groups than in the normoxia group (Figure 3A). Similarly, velocity was significantly lower in the chemical (*P* = .007) and gas (*P* = .03) treatment groups than in the normoxia group at 2 hours post‐treatment (Figure 3B). Time in motion did not differ between the normoxic group and either the chemical (*P* = .115) or gas (*P* = .09) hypoxia groups at 2 hours post‐treatment (Figure 3C).

**FIGURE 4:**
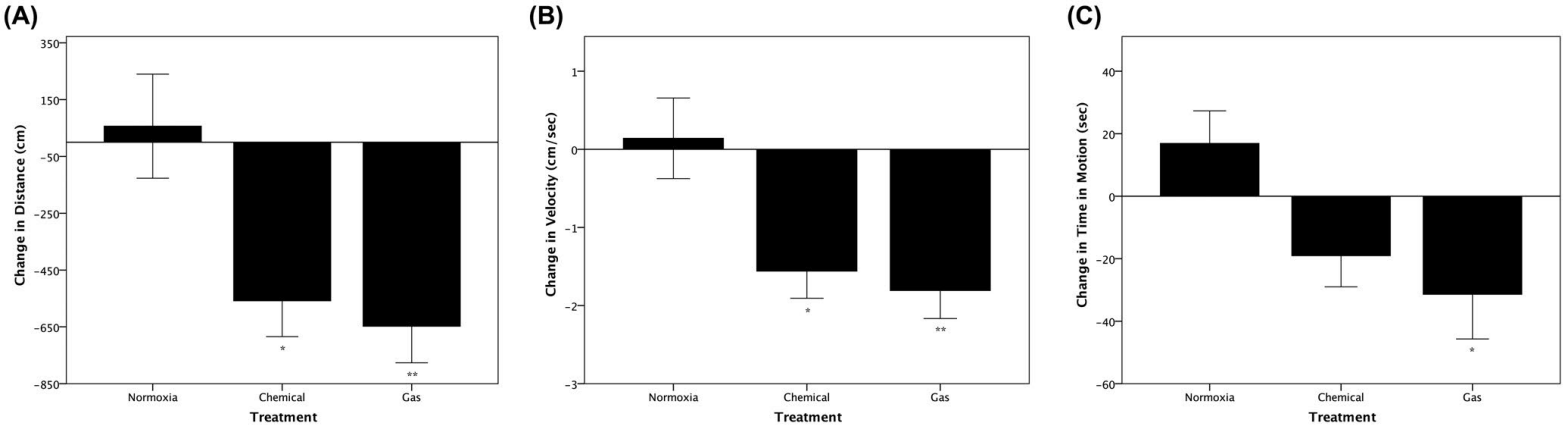
Changes in locomotor behaviors 2 hours following chemical vs nitrogen gas hypoxia. Distance travelled (A), velocity (B) and movement time (C) significantly decreased at 2 hours post‐treatment in fish exposed to either 5‐minute chemical or 10‐minute nitrogen gas hypoxia, compared to values measured in the same fish prior to hypoxia. Locomotion was recorded over a 6‐minute period while fish swam individually in observation tanks. One‐way ANOVA were conducted to determine whether changes in distance (*F*(2, 30) = 6.618; *P* = .004), velocity (*F*(2, 30) = 6.506; *P* = .004), and time in motion (*F*(2, 30) = 4.333; *P* = .022) were different for hypoxia‐treated groups vs the normoxia‐treated group. A Tukey post‐hoc analysis revealed that the difference between chemical hypoxia and normoxia was significant for distance (*P* = .015), velocity (*P* = .016), but not for time in motion (*P* = .095). It also revealed that the difference between gas hypoxia and normoxia was significant for distance (*P* = .006), velocity (*P* = .006), and time in motion (*P* = .021)

Mean changes in distance travelled, velocity, and time in motion were also calculated. One‐way ANOVAs were conducted to determine whether these changes from pre‐hypoxia to 2‐hour post‐hypoxia differed between treatment groups (Figure 4). Statistically significant differences between treatment groups were detected for changes in distance (*F*(2, 30) = 6.618, *P* = .004, *ω* ^2^ = 0.254), velocity (*F*(2, 30) = 6.506, *P* = .004, *ω* ^2^ = 0.25), and time in motion (*F*(2, 30) = 4.333, *P* = .022, *ω* ^2^ = 0.168). Tukey’s post‐hoc analysis revealed the changes in distance travelled between the *Normoxia* group and both the *Chemical* (614.7, 95% CI (107.2‐1122.2), *P* = .015) and the *Gas* (704.3, 95% CI (186.4‐1222.2), *P* = .006) hypoxia groups were significantly different (Figure 4A). The changes in velocity between the *Normoxia* group and both the *Chemical* (1.7, 95% CI (0.3‐3.1), *P* = .016) and the *Gas* (1.9, 95% CI (0.5‐3.4), *P* = .006) hypoxia groups were also significantly different (Figure 4B). The change in time in motion between the *Normoxia* group and *Gas* (48.3, 95% CI (6.4‐90.2), *P* = .021) hypoxia group was significant, but the difference between the *Normoxia* and *Chemical* groups (35.9, 95% CI (−5.1 to 77), *P* = .095) was not (Figure 4C).

### 3.4 Locomotor behaviors with 24‐hour recovery

Previous reports suggested that the locomotor effects of hypoxia would dissipate by 24 hours post‐treatment.^15^, ^24^ As with fish assessed after 2 hours of recovery, those assessed at 24 hours were divided into three groups: normoxia, chemical hypoxia, and gas hypoxia. The main objective of measurements made at 24 hours was to determine whether fish exposed to chemical hypoxia also recovered locomotor functions over that duration.

**FIGURE 5:**
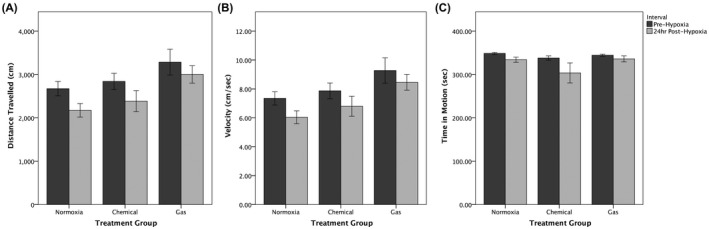
Locomotor behaviors immediately before and 24 hours after chemical vs nitrogen gas hypoxia. Zebrafish were recorded for 360 seconds prior to treatment and recorded again at a post‐treatment interval of 24 hours. *Normoxia* subjects were not exposed to hypoxia (n = 8), *Chemical* subjects were exposed to 5‐minute hypoxia caused by dissolved sodium sulfite (0.5 g/L; n = 15), and *Gas* subjects were exposed to 10‐minute hypoxia caused by bubbling with nitrogen gas (n = 11). Data are mean ± standard error. No statistically significant interactions were detected between treatment group and recording interval for (A) distance travelled (*F*(2, 31) = 0.12, *P* = .887), (B) velocity (*F*(2, 31) = 0.061, *P* = .941), or (C) time in motion (*F*(2, 31) = 0.559, *P* = .577). Additionally, no difference was detected for the effect of time or the effect of treatment group on any of the variables measured (*P* > .05)

The effects of treatment group and interactions between treatment and recording interval (pre‐hypoxia or 24‐hr post‐hypoxia) were examined by a two‐way mixed ANOVA. No statistically significant interactions between the treatment group and observation interval on distance (*F*(2, 31) = 0.120, *P* = .887, partial *η* ^2^ = 0.008), velocity (*F*(2, 31) = 0.061, *P* = .941, partial *η* ^2^ = 0.004), or time in motion (*F*(2, 31) = 0.559, *P* = .577, partial *η* ^2^ = 0.035) were detected (Figure 5). 24 hours post‐hypoxia, distance travelled was not significantly different in chemical (*P* = .802) and gas (*P* = .066) treatment groups vs the normoxia group (Figure 5A). Similarly, velocity was not significantly different in chemical (*P* = .701) and gas (*P* = .056) treatment groups vs the normoxia group at 24 hours post‐treatment (Figure 5B). Time in motion did not differ between the normoxic group and either the chemical (*P* = .507) or gas (*P* = .998) hypoxia groups at 24 hours post‐treatment (Figure 5C).

**FIGURE 6:**
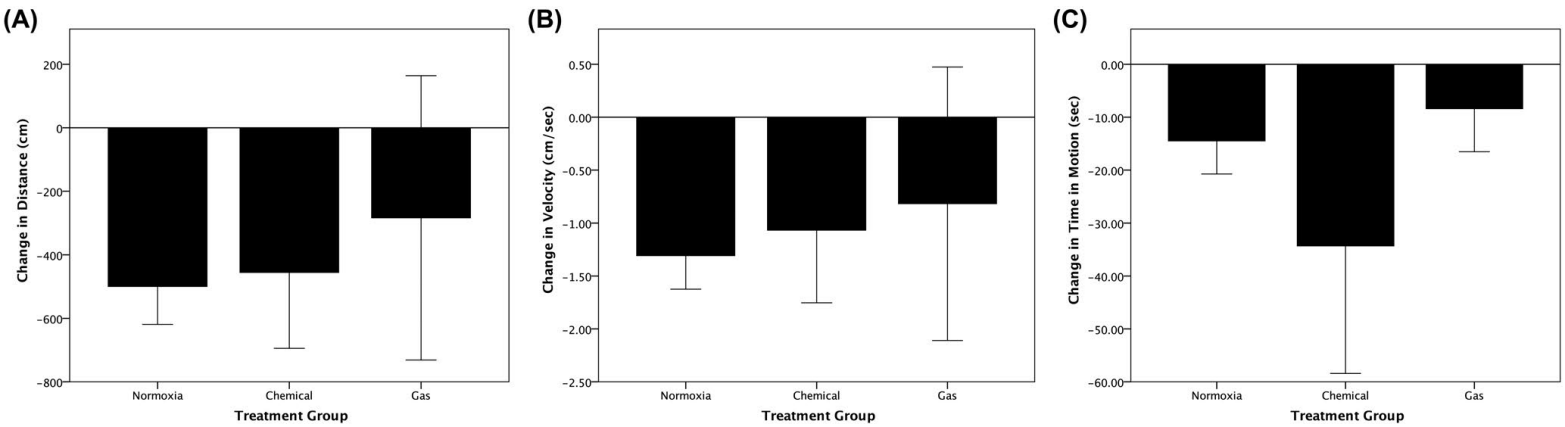
Changes in locomotor behaviors 24 hours following chemical vs nitrogen gas hypoxia. Locomotion was recorded over a 6‐minute period while fish swam individually in observation tanks at two timepoints: immediately prior to treatment and at 24 hours after treatment. The difference in measurement between those two timepoints was calculated and is reported here. One‐way ANOVA were conducted to determine whether changes in distance (*F*(2, 31) = 0.120; *P* = .887), velocity (*F*(2, 31) = 0.061; *P* = .941), and time in motion (*F*(2, 31) = 0.559; *P* = .577) were different for hypoxia‐treated groups vs the normoxia‐treated group. No significant differences were detected

Mean changes in distance travelled, velocity, and time in motion were also calculated. One‐way ANOVAs were conducted to determine whether these changes from pre‐hypoxia to 24‐hour post‐hypoxia differed between treatment groups (Figure 6). Statistically significant differences between treatment groups were not detected for changes in distance (*F*(2, 31) = 0.120, *P* = .887, *ω* ^2^ = 2 × 10^−11^), velocity (*F*(2, 31) = 0.061, *P* = .941, *ω* ^2^ = 3 × 10^−6^), or time in motion (*F*(2, 31) = 0.559, *P* = .577, *ω* ^2^ = 0). These results are consistent with previous findings that the locomotor effects of hypoxia are no longer present 24 hours after treatment.

## 4 DISCUSSION

For nearly a decade, zebrafish models have been used to examine the mechanisms of hypoxic‐ischemic damage in the nervous system. Initial studies established nitrogen gas bubbling as a method for creating a hypoxic environment in aquarium water.^13^ Hypoxia recovery was further studied with this model in the presence of zinc chelator, DEDTC.^22^ In that study, Yu and Li reported that exposure to the zinc chelator–reduced sensitivity to hypoxia in their subjects. Their experimental design helped to establish the use of the zebrafish model for pharmacologic studies of hypoxic‐ischemic damage.

At around the same time, another group showed that simple locomotor assays could be used to quantify the effects of hypoxic injury in zebrafish.^15^ They found that movement was slowed for up to 6 hours following exposure to nitrogen gas hypoxia. They also reported that locomotor deficits were no longer observed 24 or 48 hours after hypoxia.

Our goals for this study were to minimize subject mortality, maximize hypoxia reproducibility, and match behavioral outcomes from these previous reports. Minimizing subject mortality while maintaining quantifiable outcomes is vital to the utility of this zebrafish model of hypoxia‐ischemia. Previous studies reported mortality rates of 60% or more.^13^ Such low survival would slow progress in behavioral studies that require large numbers of subjects and could serve as a barrier to the use of genetically modified zebrafish; it would also greatly increase the number of subjects required for all studies. It is therefore important to note that mortality did not exceed 12% with our methods.

The reduction in mortality is owed, at least in part, to an adjustment made to our hypoxia chamber. By eliminating any air gaps within the chamber, we prevented aquatic surface respiration, a behavior seen in zebrafish in response to hypoxia.^26^ This seemed to reduce variability in the progression of the behavioral response to hypoxia between fish. We were thus able to simplify our protocol by exposing fish to hypoxia for a set duration rather basing duration on behavioral observations, as described previously.^15^ Although we were concerned that applying a set duration of hypoxia rather than choosing the duration based on behavioral responses could increase the variability in our data, this did not seem to be the case. Eliminating air gaps in the hypoxia chamber and exposing to a set duration of hypoxia are therefore two ways to maximize reproducibility between researchers and between subjects.

In addition to matching the locomotor effects of hypoxia created by bubbling with nitrogen gas, hypoxia created by sodium sulfite provided benefits. One benefit was a reduction in hypoxia duration required for locomotor effects. Fish were immobilized by chemical hypoxia at 5 minutes, whereas 10 minutes passed before immobilization by nitrogen gas hypoxia. This may be explained by the significantly lower dissolved oxygen concentrations obtainable by sodium sulfite treatment vs nitrogen gas (Figure 1C). A related benefit was a measured reduction in DO variation with chemical hypoxia (SD = ±0.08 mg/L) vs gas hypoxia (SD = ±0.18 mg/L), suggesting that hypoxia by sodium sulfite is more reproducible than by nitrogen gas. A third benefit is not shown in the data. The use of nitrogen gas for hypoxia requires control of compressed gas flow that can be difficult to maintain at constant pressures. This becomes a challenge at undergraduate institutions where novice researchers may only work on a project for a matter of months. In addition, the addition of sodium sulfite to aquarium water requires minimal training and is easily reproduced by researchers. Therefore, in addition to producing lower mean dissolved oxygen concentrations and reduced variability, sodium sulfite also offers a simpler method for inducing hypoxia in aquarium water.

## CONFLICT OF INTEREST

The authors have declared no conflicts of interest for this article.

## AUTHOR CONTRIBUTIONS

JW conceived and designed the study. KM and ES helped to develop the final methodology, performed experiments, and collected data. JW and KM performed data analysis. JW drafted the manuscript and all authors contributed to manuscript editing and approval.
